# Adherence to the Mediterranean Diet and Risk of Metabolically Unhealthy Obesity in Women: A Cross-Sectional Study

**DOI:** 10.3389/fnut.2022.858206

**Published:** 2022-04-25

**Authors:** Alessandro Leone, Ramona De Amicis, Alberto Battezzati, Simona Bertoli

**Affiliations:** ^1^International Center for the Assessment of Nutritional Status, Department of Food, Environmental and Nutritional Sciences, University of Milan, Milan, Italy; ^2^Lab of Nutrition and Obesity Research, Istituto Auxologico Italiano, IRCCS, Milan, Italy

**Keywords:** metabolically healthy obesity, obesity phenotypes, mediterranean diet, metabolic syndrome, women

## Abstract

Some obese individuals do not present any metabolic alteration and are considered metabolically healthy (MHO). Adherence to high-quality dietary pattern may favor this phenotype. We aimed to evaluate the association between the adherence to the Mediterranean diet and risk of metabolically unhealthy obesity (MUO) in women. We conducted a cross-sectional study on 2,115 obese women. All patients underwent a medical examination, anthropometric evaluation, bioelectrical impedance, ultrasound measurements of abdominal visceral (VAT) and subcutaneous (SAT) fat, blood sampling and evaluation of adherence to the Mediterranean diet through MEDAS questionnaire. The diagnosis of MHO and MUO was made using the harmonized criteria. A multivariable logistic regression adjusted for age, BMI, fat free mass, ultrasound-estimated VAT:SAT ratio, marital status, education, past diet, antidepressant use, family history of diabetes and cardiovascular disease, menopausal status, smoking, and physical activity was used to assess the association between Mediterranean diet and MUO risk. The prevalence of MHO was 21.2% (*N* = 449). Compared to MUO women, MHO women were younger, had lower BMI and VAT, and had higher fat free mass and SAT. In the multivariable model, the adherence to the Mediterranean diet was not associated with the risk of MUO (OR = 0.91, 95%CI: 0.62; 1.34, *P* = 0.624). Given the impact of menopause on metabolic health we also carried out the analysis in pre- and post-menopausal women separately. Higher adherence to the Mediterranean diet was associated with a lower risk of MUO in postmenopausal women (OR = 0.55, 95%CI: 0.31; 0.96, *P* = 0.034). No association was found in premenopausal women (OR = 1.18, 95%CI: 0.70; 1.99, *P* = 0.532). In conclusion, adherence to the Mediterranean diet was associated with a better metabolic health in postmenopausal women. Further studies are needed to confirm the ability of the Mediterranean diet in promoting maintenance of the healthy phenotype and reversion from MUO.

## Introduction

Obesity is a complex, multifactorial disease that affects 13% of the population worldwide (1). It represents a major public health problem, with a significant impact on health and national health expenditure (2). Obese people have an increased risk for many metabolic complications and chronic diseases, such as type 2 diabetes, cardiovascular disease, and some forms of cancer, as well as a reduction in life expectancy (3, 4). Moreover, recent evidence has led to the conclusion that obesity is a risk factor for the communicable disease COVID-19 (5) and might also promote vaccine-breakthrough SARS-CoV-2 infections in fully vaccinated people (6). Despite this, some obese individuals do not present metabolic abnormalities and are therefore considered metabolically healthy (MHO) (7). A meta-analysis of 12 cohort studies and 7 intervention studies found that 35% of obese people were metabolically healthy, with significant differences among countries (8). In Italy, the prevalence of MHO ranged from 11.5 to 29.2%, with higher rates in women (9). Several studies have shown that MHO individuals have a lower risk of developing type 2 diabetes, cardiovascular disease and premature death than individuals with a metabolically unhealthy phenotype (MUO) (10–12). Adipose tissue plays a central role in defining the metabolic phenotype of obesity (13). The expandability of subcutaneous adipose tissue, particularly in the gluteofemoral region is considered beneficial for metabolic health and preservation of insulin sensitivity. On the other hand, MUO individuals are characterized by adipocyte hypertrophy, increased visceral and subcutaneous abdominal adipose tissue, and increased ectopic lipid deposition in organs such as liver and skeletal muscle (14). This hypertrophic adipose tissue is also characterized by increased macrophage infiltration, inflammation, and altered adipokines secretion. Together, these disturbances may lead to the development of peripheral insulin resistance (15). However, it must be said that metabolic health is a transient state for a large proportion of women with obesity (16). With aging, in fact, there is a decline in the capacity of subcutaneous adipose tissue to respond to overfeeding by hyperplasia and a redistribution of adipose tissue. In women, the turning point is represented by menopause, during which the largest redistribution of adipose tissue from the gluteofemoral region to the abdominal region occurs, with consequent increased cardiometabolic risk (14).

Because MHO individuals have a lower risk of cardiometabolic disease than individuals with unhealthy obesity, it is important to promote the transition from the unhealthy to the healthy phenotype (17). In people with obesity, lifestyle intervention aimed at weight loss is considered the first line intervention. Weight loss promotes a reduction in cardiometabolic risk and also provides benefits for other obesity-related issues. However, the amount of weight loss required to facilitate the transition from MUO to MHO is still a matter of debate. Some evidence suggests that the greater the starting BMI and the amount of liver fat, the greater the weight loss will need to be (17). However, weight loss is not the only target that can promote the transition from MUO to MHO. There is substantial evidence that a healthy lifestyle can reduce the cardiometabolic risk, independent of effects on bodyweight (17). Adherence to high-quality dietary patterns is known to be crucial in reducing the risk of obesity-related comorbidities (18). Among high-quality dietary patterns, the Mediterranean diet has been associated with lower weight gain (19–22), obesity prevention (23), reduced cardiovascular risk also in obesity (24), reversion and lower risk of metabolic syndrome (25), more favorable inflammatory status (26), and better distribution of abdominal adipose tissue (27, 28). On the basis of these findings, we hypothesized that adherence to the Mediterranean diet in obesity could be associated with metabolic health. However, evidence on this topic is still limited, and therefore, to test our hypothesis, we assessed the adherence to the Mediterranean diet in a sample of obese women who were then classified as MHO and MUO.

## Materials and Methods

### Study Design

We conducted a cross-sectional study on patients recruited at the International Center for the Assessment of Nutritional Status (ICANS), a nutritional outpatient clinic of the University of Milan that started its clinical activity in 2003. The promotion of the clinical activity took place, initially, through the use of flyers and newspaper advertisement, and then through a web page. Patients came to ICANS on a voluntary basis or sent by their general practitioner or a specialist. The objective was to perform a nutritional assessment aimed at obtaining a personalized dietary program for weight loss and/or improvement of metabolic parameters, or to perform specific instrumental and/or laboratory evaluations. The data collected were entered into an electronic medical record and, after obtaining a signed informed consent, conveyed into a large database and used, in an anonymized manner, for research purposes.

For the study, we selected obese women (BMI ≥ 30 kg/m^2^), aged ≥18 years, not previously diagnosed with type 1 and 2 diabetes and cardiovascular disease, not having neurological, gastrointestinal, cardiac, renal, and pulmonary failure, cancer in the last 5 years, and acute illness, not using drugs known to cause lipodystrophy including steroids and antiretroviral agents, and come to ICANS before the COVID-19 pandemic. Up to February 2020, 2,757 obese women with the characteristics described above had been recruited. To be eligible for the study, all women should have been subjected to a medical examination, anthropometric evaluation, bioelectrical impedance, ultrasound measurements of abdominal fat, blood sampling and evaluation of adherence to the Mediterranean diet through a dietary screener. Therefore, we excluded 507 women who had performed only specific instrumental and/or laboratory evaluations, and 135 women who did not complete (≥1 item missing) the dietary screener for the assessment of the adherence to the Mediterranean diet. The characteristics of excluded women are given in Supplementary Table 1. Women included in the study were significantly younger, especially compared with women excluded because they had one or more missing items in the MEDAS questionnaire. The latter had less fat free mass (FFM) and greater abdominal visceral fat (VAT) thickness, as well as being less educated.

The study was conducted following the guidelines laid down in the Declaration of Helsinki and the Ethics Committee of the University of Milan gave a positive opinion on study procedures (protocol no. 23/2016).

### Measurements

On arrival at ICANS, the patients completed a short questionnaire investigating socio-demographic information. Between 08:30 and 09:00 AM, a physician took a blood sample from the fasting patient. The sample was then centrifuged and an aliquot of serum was used for the determination of blood glucose, triglycerides and HDL-cholesterol. The above biochemical parameters were measured by enzymatic method (Cobas Integra 400 Plus, Roche Diagnostics, Rotkreuz, Switzerland). The physician then conducted a medical examination to obtain information about any past dietary interventions, patient’s medical history, family history of diabetes and cardiovascular disease, lifestyle, with particular regard to smoking and weekly structured physical activity, and menopausal status. Menopause has been defined as the absence of a menstrual cycle for at least 12 months. The physician performed an ultrasound measurement of abdominal visceral and subcutaneous (SAT) fat using a Logiq 3 Pro instrument equipped with a 7.5 MHz linear probe and with a 3.5 MHz convex-array probe (GE Healthcare, Chicago, IL, United States). The measurements were taken 1 cm above the umbilicus at the end of expiration. SAT, measured with the 7.5 MHz linear probe, was defined as the distance between the epidermis and the external face of the rectus abdominis muscle; VAT, measured with the 3.5 MHz convex-array probe, was defined as the distance between the anterior wall of the aorta and the posterior surface of the rectus abdominis muscle (29, 30). The physician then performed systolic and diastolic blood pressure measurements in accordance with JNC-7 guidelines (31).

A registered dietician took the anthropometric measurements according to international guidelines (32) and performed body composition assessment by bioelectric impedance. Weight was taken with an electronic scale and rounded to the nearest 100 g (Seca 700 balance, Seca Corporation). Height was measured with a vertical stadiometer with an accuracy of 0.1 cm. Body mass index (BMI) was then calculated and classified using WHO cut-offs (33). Waist circumference (WC) was measured with a non-elastic tape at the midpoint between the last rib and the iliac crest with an accuracy of 0.5 cm. Body composition was assessed using a tetra polar 8-point tactile electrode system (InBody 720, Biospace, Seoul, South Korea) at 1, 5, 50, 250, 500, and 1,000 kHz. Participants stood on the scale platform of the instrument and grasped the handles of the device, to provide contact with a total of eight electrodes (two for each foot and for each hand). Manufacturer’s equations were used to estimate total body fat and FFM. All measurements were taken with the patient wearing only light clothes.

### Outcome Assessment

The diagnosis of MHO and MUO was made using the harmonized criteria proposed by Lavie et al. (34) (Table 1). Briefly, a women was classified as MHO if she met no criteria for metabolic syndrome (MetS), with the exclusion of high WC. Conversely, a women was classified as MUO if she met 1 to 4 MetS criteria (WC excluded).

**TABLE 1:** Diagnostic criteria for metabolic phenotypes of obesity.

Definition of MHO	
A women has been classified as MHO if **met 0 of the 4 MetS criteria** (WC	
excluded), which are the following:	
• Elevated triglycerides or drug treatment for elevated triglycerides	≥150 mg/dl (1.7 mmol/l)
• Reduced high-density lipoprotein cholesterol or drug treatment for reduced HDL	<50 mg/dl (1.3 mmol/l)
• Elevated blood pressure or antihypertensive drug treatment	Systolic blood pressure ≥ 130 mm Hg and/or diastolic blood pressure ≥ 85 mm Hg
• Elevated fasting glucose or drug treatment of elevated glucose	≥100 mg/dl (5.6 mmol/l)

**Definition of MUO**	

A women has been classified as MUO if **met 1 to 4 of the MetS criteria**	
reported above (WC excluded).	

*MHO, metabolically healthy obesity; MUO, metabolically unhealthy obesity; MetS, metabolic syndrome; WC, waist circumference; and BP, blood pressure.*

### Exposure Assessment

During the morning, patients completed the MEDiterranean Diet Adherence Screener (MEDAS) (35), a 14-item questionnaire developed in the PREDIMED trial. The questionnaire investigates some dietary habits, the consumption of typical foods of the Mediterranean diet (oil, vegetables, fruit, wine, legumes, fish, nuts, white meat, and use of soffritto) and the consumption of unhealthy foods (red and processed meat, animal fat, sugary and carbonated beverages and sweets). For each item a 1 point is given if the consumption meets the criteria of adherence to the Mediterranean diet (Table 2). The sum of the individual points gives a Mediterranean score, which is a number between 0 and 14. A score ≥9 is considered an indication of adherence to the Mediterranean diet (36–38).

**TABLE 2:** Questions and criteria for assessing the adherence to the Mediterranean diet.

MEDAS question	Criteria for 1 point
1. Do you use olive oil as the principal source of fat for cooking?	Yes
2. How much olive oil do you consume per day (including that used in frying, salads, meals eaten away from home, etc.)?	≥4 table spoons/day
3. How many servings of vegetables do you consume per day?	≥2 servings/day
4. How many pieces of fruit (including fresh-squeezed juice) do you consume per day?	≥3 servings/day
5. How many servings of red meat, hamburger, or sausages do you consume per day?	<1 serving/day
6. How many servings (12 g) of butter, margarine, or cream do you consume per day?	<1 serving/day
7. How many carbonated or sugar-sweetened beverages do you consume per day?	<1 serving/day
8. Do you drink wine? How much do you consume per week?	≥3 glasses/week
9. How many servings of pulses do you consume per week?	≥3 servings/week
10. How many servings of fish/seafood do you consume per week?	≥3 servings/week
11. How many times do you consume commercial (not homemade) pastry such as cookies or cake per week?	<3 times/week
12. How many times do you consume nuts per week?	≥1 servings/week
13. Do you prefer to eat chicken, turkey, or rabbit instead of beef, pork, hamburgers, or sausages?	Yes
14. How many times per week do you consume dishes prepared soffritto?	≥2 servings/week

### Statistical Analysis

Continuous variables are reported as median and interquartile range (IQR), as many of them did not follow a normal distribution. Discrete variables are reported as frequency and percentage. Mann-Whitney test and Chi-squared test were used to compare distributions and proportions, respectively. A logistic regression model was fitted to assess the association between adherence to the Mediterranean diet and metabolic phenotype of obesity. Odd ratios (OR) and respective confidence intervals (CI) were calculated including the metabolic phenotype of obesity as a dependent variable (0 = MHO and 1 = MUO), and adherence to the Mediterranean diet as an independent variable (0 = non-adherent and 1 = adherent). To control for potential confounders, we used a pre-specified multivariate model, selecting variables on the basis of biological plausibility. Results were adjusted for age (continuous), BMI (continuous), FFM (continuous), ultrasound-estimated VAT:SAT ratio (continuous), marital status (unmarried, married, widower/separated), education (elementary or middle school, high school, master degree or higher), past diet (yes, no), antidepressant use (yes, no), family history of diabetes (yes, no) and cardiovascular disease (yes, no), menopausal status (premenopausal, postmenopausal), smoking (never, former, current), and physical activity (no, at least 2 h/week). No evidence of multicollinearity was found. The linearity of continuous variables was tested using multivariable fractional polynomials. Following this approach, we found that using untransformed variables ensured better fits for the model. We assessed the goodness of fit (GOF) of the models using the standardized Pearson test. In view of the available knowledge, we consider this to be a sufficient proof of the acceptable fit of the model (39). A *p*-value of <0.05 was considered statistically significant. Statistical analysis was performed using STATA version 12.0 (StataCorp, College Station, TX, United States).

## Results

We included 2,115 obese women with a median age of 49 years (IQR: 40; 58 years) and a median BMI of 33.3 kg/m^2^ (IQR: 31.4; 36.3 kg/m^2^). Fifteen percent of the women had previously followed a dietary program aimed at weight loss, and 36% reported to be engaged in structured physical activity for at least 2 h/week. Women who followed a diet in the past were more physically active (69%) than women who never followed a diet program to lose weight (31%, *p* < 0.001). Supplementary Table 2 gives an overview of the food consumption of recruited women.

Table 3 shows the characteristics of the patients recruited for the study according to metabolic phenotype. In our sample, 449 (21.2%) women were free of metabolic abnormality and were classified as MHO, while the remaining 78.8% of women had at least one metabolic alteration and were classified as MUO. Compared to MUO women, MHO women were younger. Prevalence of MHO decreased with each decade (38.0% at 18–29 years, 37.8% at 30–39 years, 25.6% at 40–49 years, 13.2% at 50–59 years, 7.2% at 60–69 years and 3.4% at ≥70 years; *P* < 0.001). MHO women had lower BMI and ultrasound-estimated VAT and a higher FFM and ultrasound-estimated SAT than MUO women. No difference between the two groups was observed regarding adherence to the Mediterranean diet. Overall, adherence to the Mediterranean diet was found in 264 women (11.6%). As expected, adherence to the Mediterranean diet was influenced by age (6.7% at 18–29 years, 7.8% at 30–39 years, 7.3% at 40–49 years, 11.8% at 50–59 years, 21.6% at 60–69 years and 17.8% at ≥70 years; *P* < 0.001).

**TABLE 3:** Characteristics of patients.

	MHO *N* = 449		MUO *N* = 1666		
	Median	IQR	Median	IQR	*P*-value
Age (years)	41	34; 49	51	42; 60	<0.001
BMI (kg/m^2^)	32.3	31.0; 34.1	33.7	31.6; 36.8	<0.001
Fat Free Mass (%)	59.3	57.5; 61.0	57.3	55.3; 59.1	<0.001
Waist circumference (cm)	100.5	96.0; 105.7	105.6	100.2; 112.0	<0.001
Visceral fat (mm)	45.3	35.0; 58.2	63.7	48.5; 80.6	<0.001
Subcutaneous fat (mm)	36.4	29.1; 43.5	33.9	26.9;42.1	<0.001
VAT:SAT ratio	1.2	0.9; 1.8	1.8	1.3; 28	<0.001
Triglycerides (mg/dl)	75	59; 97	108	80; 148	<0.001
HDL cholesterol (mg/dl)	63	59; 71	56	48; 66	<0.001
Serum glucose (mg/dl)	90	86; 94	98	91; 105	<0.001
Systolic blood pressure (mm Hg)	120	110; 120	130	120; 140	<0.001
Diastolic blood pressure (mm Hg)	75	70; 80	80	80; 85	<0.001
Mediterranean score	6	5; 7	7	5; 8	0.009

	**N**	**%**	**N**	**%**	

**Marital status**					
Not married	194	43.2	510	30.6	<0.001
Married	215	47.9	961	57.7	
Divorced or widower	40	8.9	195	11.7	
**Education**					
Elementary or middle school	56	12.5	255	15.3	0.262
High school	239	53.2	883	53	
Master degree or higher	154	34.3	528	31.7	
**Smoking**					
Not smoker	261	58.1	925	55.5	0.613
Ex-smoker	86	19.2	341	20.5	
Smoker	102	22.7	400	24	
**Structured physical activity**					
No	286	63.7	1056	63.4	0.903
At least 2 h/week	163	36.3	610	36.6	
**Menopausal status**					
Premenopausal	347	77.3	740	44.4	<0.001
Postmenopausal	102	22.7	926	55.6	
**Familiarity for diabetes**					
No	291	64.8	1047	62.8	0.443
Yes	158	35.2	619	37.2	
**Familiarity for CVD**					
No	299	66.6	1143	68.6	0.416
Yes	150	33.4	523	31.4	
**Cancer**					
Healed for at least 5 years	23	5.1	116	7.0	0.163
Never been diagnosed	426	94.9	1550	93.0	
**Past dietary program**					
No	386	86	1407	84.5	0.428
Yes	63	14	259	15.5	
**Using antidepressants**					
No	426	94.9	1565	93.9	0.452
Yes	23	5.1	101	6.1	
**Treatment for high triglycerides**					
No	449	100	1525	91.5	<0.001
Yes	0	0.0	141	8.5	
**Treatment for low HDL cholesterol**					
No	449	100	1664	99.9	0.463
Yes	0	0.0	2	0.1	
**Treatment for high blood pressure**					
No	449	100	1226	73.6	<0.001
Yes	0	0.0	440	26.4	
**Adherence to the Mediterranean diet**					
Not adherent	404	90.0	1470	88.2	0.302
Adherent	45	10.0	196	11.8	

When we studied the association between adherence to the Mediterranean diet and the risk of MUO (Table 4), we found no association in the multivariable model (OR = 0.91, 95%CI: 0.62; 1.34, *P* = 0.624). Given the impact of menopause on metabolic health we also carried out the analysis in pre- and post-menopausal women separately. We found that following a Mediterranean diet was associated with a lower risk of MUO in postmenopausal women (OR = 0.55, 95%CI: 0.31; 0.96, *P* = 0.034). No association was found between adherence to the Mediterranean diet and risk of MUO in premenopausal women (OR = 1.18, 95%CI: 0.70; 1.99, *P* = 0.532).

**TABLE 4:** Association between the adherence to the Mediterranean diet risk of metabolically unhealthy obesity.

		Adherence to the		
		Mediterranean diet		
		Not adherent	Adherent	*P*-value
Overall	MHO/MUO	404/1470	45/196	
	Median score	6	9	
	OR (95%CI)	1 (ref.)	0.91 (0.62; 1.34)	0.624
Premenopausal women	MHO/MUO	321/683	26/57	
	Median score	6	9	
	OR (95%CI)	1 (ref.)	1.18 (0.70; 1.99)	0.532
Postmenopausal women	MHO/MUO	83/787	19/139	
	Median score	7	9	
	OR (95%CI)	1 (ref.)	0.55 (0.31; 0.96)	0.034

*Models adjusted for age, BMI, fat free mass (%), VAT:SAT ratio, past diet, marital status, education, smoking, physical activity, menopausal status, familiarity for diabetes and cardiovascular disease and antidepressants use.*

Table 5 show the adjusted ORs in sensitivity analysis after modifying some our assumptions. The results did not change when we included women with one or more missing items in the MEDAS questionnaire and after exclusion of women who followed a structured dietary program aimed at weight loss in the past, taking antidepressants and declared cured of cancer for at least 5 years. When we included women with missing information on body composition (ultrasound-estimated VAT and SAT and/or FFM) the statistical significance was marginally lost (*P* = 0.068) in postmenopausal women. Note, however, that to conduct the latter analysis, we had to remove the variables of body composition and abdominal fat distribution from the confounders.

**TABLE 5:** Sensitivity analysis.

		Adherence to the Mediterranean diet		
	MHO/MUO	Not adherent	Adherent	*p*-value
**Overall**				
Including women with missing item in the MEDAS questionnaire	467/1783	1 (ref.)	0.91 (0.62, 1.35)	0.648
Including women without body composition assessment*	484/1793	1 (ref.)	0.86 (0.59, 1.25)	0.429
Excluding women taking antidepressants	426/1565	1 (ref.)	0.92 (0.61, 1.38)	0.686
Excluding women with past cancer	426/1550	1 (ref.)	0.88 (0.59, 1.32)	0.542
Excluding women following a diet in the past	404/1513	1 (ref.)	0.90 (0.58, 1.38)	0.621
**Premenopausal women**				
Including women with missing item in the MEDAS questionnaire	361/773	1 (ref.)	1.22 (0.73, 2.04)	0.455
Including women without body composition assessment*	377/801	1 (ref.)	1.06 (0.64, 1.75)	0.828
Excluding women taking antidepressants	332/696	1 (ref.)	1.23 (0.72, 2.12)	0.445
Excluding women with past cancer	337/711	1 (ref.)	1.13 (0.67, 1.90)	0.640
Excluding women following a diet in the past	325/702	1 (ref.)	1.21 (0.70, 2.10)	0.498
**Postmenopausal women**				
Including women with missing item in the MEDAS questionnaire	106/1010	1 (ref.)	0.54 (0.31, 0.94)	0.030
Including women without body composition assessment*	107/992	1 (ref.)	0.61 (0.35, 1.04)	0.068
Excluding women taking antidepressants	94/869	1 (ref.)	0.56 (0.31, 0.99)	0.049
Excluding women with past cancer	89/839	1 (ref.)	0.51 (0.28, 0.92)	0.025
Excluding women following a diet in the past	79/811	1 (ref.)	0.48 (0.26, 0.90)	0.022

*Models adjusted for age, BMI, fat free mass (%), VAT:SAT ratio, past diet, marital status, education, smoking, physical activity, menopausal status, familiarity for diabetes and cardiovascular disease, and antidepressants use.*

*model without fat free mass (%) and VAT:SAT ratio.

## Discussion

In this study, the adherence to the Mediterranean diet was favorably associated with metabolic phenotype of obesity in older women. More specifically, the adherence to the Mediterranean diet was associated with a lower likelihood of MUO in postmenopausal women, independent of wide range on known confounders. Moreover, the result appears robust, such that sensitivity analysis did not show major losses of statistical significance regarding the association between adherence to the Mediterranean diet and metabolic health in postmenopausal women. Only when we included women with missing information on body composition and abdominal fat distribution, the association was marginally lost. However, in the latter case we had to remove these important confounders from the analysis, and this may explain the marginal loss of significance.

Metabolically healthy obesity is a phenotype of obesity characterized by the absence of metabolic alterations. In agreement with previous epidemiological studies, approximately one in five obese women in our sample had this metabolic phenotype (9, 13), but the prevalence was influenced by age. Our results confirm the differences in body composition and adipose tissue distribution between the two metabolic phenotypes of obesity (15). Women with the healthy phenotype had higher FFM and ultrasound-estimated SAT, whereas women with the unhealthy phenotype had greater amount of fat mass and ultrasound-estimated VAT. Previous epidemiological studies report similar caloric and nutrient intakes, as well as equal consumption of different food groups, among MHO and MUO individuals (40–42). However, studying the dietary pattern may better inform the holistic effect of diet on healthy obesity (42). Assessment of dietary patterns avoids potential confounding with other aspects of the diet, increases the ability to assess stronger effects due to the cumulative effects of many dietary characteristics, and allows assessment of the interaction between synergistic components (43–46). Indeed, current evidence, although still limited, suggests that adherence to high-quality dietary patterns, may positively affect the metabolic phenotype of obesity (47). Our results confirm this evidence, suggesting (1) that adherence to the Mediterranean diet is associated with a lower risk of MUO, (2) that the effect of dietary pattern might be greater, with regard to obese women, in postmenopausal women, and (3) that the contribution of the Mediterranean diet to metabolic health is independent of body composition and abdominal fat distribution. In a previous study where a group of pre- and post-menopausal women underwent dietary intervention to lose weight, both groups had significant body weight loss, but only the postmenopausal women had an improvement in MetS parameters (48). In younger women, the protective action of estrogen may reduce the contribution of diet to metabolic health. Estrogen drives fat accumulation in gluteofemoral adipose tissue rather than visceral adipose tissue (49, 50), and gluteofemoral adipose tissue is thought to be protective against the negative effects of obesity (51). Estrogens also have anti-inflammatory and antioxidant properties, as they reduce the release of pro-inflammatory cytokines by immune cells, and increased resistance to oxidative stress (50, 52). Consistent with these findings, when compared with postmenopausal women, premenopausal women have been found less insulin resistance (52). The protective effect is lost with the withdrawal of estrogen after menopause. As a consequence, fat accumulation in visceral adipose tissue increases, as does the risk of developing insulin resistance and CVD (52). It is therefore possible that when estrogen protection is lost, diet becomes more relevant. An alternative explanation could be related to the low adherence to the Mediterranean diet observed in younger women. This finding confirms the abundant evidence showing that younger segments of the population are abandoning the Mediterranean dietary pattern in favor of the less healthy, but more appealing, Western dietary patterns (38, 53).

Our results find consistency in a cohort study conducted within the PREDIMED trial, where greater adherence to the Mediterranean diet, assessed through the MEDAS screening tool, was associated with transition to the healthy obesity phenotype in men and women aged 55 years and older (54). In contrast, they differed from the results of a previous cross-sectional study, where a higher Mediterranean diet score was associated with a higher likelihood of MHO in men aged <45 years and pre-menopausal women, but not in the older age groups (55). The discrepancy could lie in the different assessment tool for dietary habits, in the intrinsic differences in the studied populations (American population vs. Mediterranean population), as well as in the different definition of MHO. The lack of universally recognized criteria to define MHO makes it difficult to compare studies. The criteria proposed by Lavie et al. (34) and that we used in the present study are based on the harmonized criteria for the diagnosis of MetS (56), parameters that are readily available and widely used in rutinary clinical practice and research, making our findings, in fact, more easily comparable. Moreover, they define the MHO phenotype as free of metabolic alterations. The presence of even a single metabolic alteration is instead to be considered at risk of cardiovascular event, as the latter increases progressively with the number of metabolic alterations (57).

Several beneficial effects attributable to the Mediterranean diet could explain its role in promoting metabolic health. It has been reported from both prospective cohort studies and intervention trials how the Mediterranean diet, even in the absence of energy restriction, prevent weight gain (19, 21–23). In addition, several evidences suggest that adherence to the Mediterranean diet reduces liver fat content (58, 59), and, thereby, improves glucose and lipid metabolism, and, also *via* regulation of hepatokine release, impacts on the cardiometabolic risk (60). Finally, several bioactive compounds introduced by following a Mediterranean diet, such as polyphenols, mono and polyunsaturated fatty acids, micronutrients and antioxidants, contribute to metabolic health by reducing oxidative stress and inflammation (61, 62). On the other hand, it is presumable that women with lower adherence to the Mediterranean diet followed a diet that was higher in high-calorie foods of low nutritional quality. This may have contributed to a higher intake of refined carbohydrates and sugars, saturated fat, salt and additives, and a lower intake of fiber and micronutrients, promoting visceral fat accumulation (54) and a low-grade inflammatory state (63, 64), risk factors for developing insulin-resistance (65).

Several strengths characterize the present study. First, the large sample size. Second, we were able to control for a wide range of confounders, including body weight and composition, estimates of visceral and subcutaneous abdominal fat, education and marital status, assumed to be proxies for socioeconomic status and access to health care, family history of diabetes and cardiovascular disease, menopausal status and lifestyle. All variables known to influence metabolic status.

We are well aware, however, that the study is not without limitations. First, the cross-sectional design does not allow for a cause-effect relationship. Second, we included only women. Third, we used a short dietary screener to assess adherence to the Mediterranean diet, so a limited number of foods were considered. However, the questionnaire was shown to have a good agreement with Mediterranean diet adherence estimated by a FFQ (35). Fourth, the use of a self-completed dietary questionnaire may have been difficult for older, less educated women to understand, causing selection bias. However, exclusion of these women did not appear to affect the association between Mediterranean diet and metabolic health. Fifth, the use of non-gold-standard techniques for the assessment of body composition and abdominal adipose tissue distribution calls for caution in the interpretation of results. To this should be added that no comparison with reference methods was made in this study. In fact, we used bioelectrical impedance, which, although widely used in clinical settings, provides an estimate of the amount of FFM. We also performed an estimation of visceral and subcutaneous abdominal adipose tissue by ultrasonography, which, although some published works show good correlation between ultrasound thicknesses of VAT and SAT and the respective areas measured by computed tomography and MRI (29, 66), is not the reference technique for the evaluation of abdominal adipose tissue distribution. Sixth, our study included only Caucasian women, and, therefore, these findings cannot be transferred to women of other ethnicities without a prior confirmation. Seventh, the use of hormone replacement therapy in the years after menopause may have influenced the risk of metabolic alterations. Finally, as in any observational study, potential residual confounding could not be ruled out.

## Conclusion

In conclusion, adherence to the Mediterranean diet in obese women is associated with a better metabolic health. The contribution of diet seems to be more relevant in postmenopausal. Further prospective epidemiological studies and clinical trials are needed to confirm that the Mediterranean diet may promote maintenance of the healthy metabolic phenotype and reversion from the unhealthy phenotype.

## Data Availability Statement

The raw data supporting the conclusions of this article is available from the corresponding author on reasonable request.

## Ethics Statement

The study procedures were reviewed and approved by the Ethics Committee of the University of Milan. Patients provided their written informed consent to participate in this study.

## Author Contributions

AL: conceptualization, data curation, formal analysis, investigation, supervision, visualization, methodology, writing – original draft, and writing – review and editing. RD: investigation and writing – review and editing. AB: investigation, funding acquisition, and writing – review and editing. SB: conceptualization, investigation, funding acquisition, and writing – review and editing. All authors contributed to the article and approved the submitted version.

## Conflict of Interest

The authors declare that the research was conducted in the absence of any commercial or financial relationships that could be construed as a potential conflict of interest.

## Publisher’s Note

All claims expressed in this article are solely those of the authors and do not necessarily represent those of their affiliated organizations, or those of the publisher, the editors and the reviewers. Any product that may be evaluated in this article, or claim that may be made by its manufacturer, is not guaranteed or endorsed by the publisher.
