# Supplementary material for: Adherence to the Mediterranean Diet and Risk of Metabolically Unhealthy Obesity in Women: A Cross-Sectional Study
Source: Front Nutr. 2022 Apr 25;9:858206. doi: 10.3389/fnut.2022.858206 (PMC9084308; doi:10.3389/fnut.2022.858206)
Supplement: Supplementary file 1 [file Table_1.DOCX]

Supplementary Material

# Supplementary Tables

Table S1: Characteristics of women included and excluded from the study

|  | Included  N=2115 | | Excluded (missing information in body composition or metabolic status)  N=507* | | Excluded  (missing items in the MEDAS questionnaire)  N=135 | |  |
| --- | --- | --- | --- | --- | --- | --- | --- |
|  | Median | IQR | Median | IQR | Median | IQR | P value |
| Age (years) | 49_a_ | 40; 58 | 50_b_ | 41; 59 | 56_c_ | 46; 65 | <0.001 |
| BMI (kg/m^2^) | 33.3 | 31.4; 36.3 | 33.0 | 31.3; 35.8 | 33.5 | 32.0; 36.4 | 0.128 |
| Fat Free Mass (%) | 57.7_a_ | 55.6; 59.7 | 57.7_a_ | 55.6; 59.7 | 55.9_b_ | 54.3; 57.9 | <0.001 |
| Waist circumference (cm) | 104.5 | 99.0; 110.4 | 104.0 | 98.6; 111.0 | 105.0 | 99.7; 111.3 | 0.379 |
| Visceral fat (mm) | 59.4_a_ | 44.0; 77.2 | 60.7_a_ | 45.1; 77.1 | 67.7_b_ | 49.1; 88.3 | 0.009 |
| Subcutaneous fat (mm) | 34.4 | 27.5; 42.5 | 33.6 | 26.3; 41.7 | 34.2 | 25.5; 40.8 | 0.241 |
| VAT:SAT ratio | 1.7_a_ | 1.2; 2.6 | 1.8_a_ | 1.2; 2.5 | 1.9_b_ | 1.4; 2.9 | 0.008 |
| Triglycerides (mg/dL) | 99 | 74; 136 | 104 | 73; 144 | 99 | 77; 138 | 0.278 |
| HDL cholesterol (mg/dL) | 58 | 50; 67 | 58 | 50; 70 | 57 | 49; 66 | 0.456 |
| Serum glucose (mg/dL) | 95 | 89; 102 | 96 | 88; 103 | 98 | 90; 104 | 0.300 |
| Systolic blood pressure (mm Hg) | 125_a_ | 120; 130 | 125_a_ | 120; 135 | 130_b_ | 120; 140 | <0.001 |
| Diastolic blood pressure (mm Hg) | 80 | 70; 85 | 80 | 70; 85 | 80 | 80; 85 | 0.100 |
|  | N | % | N | % | N | % |  |
| Marital status |  |  |  |  |  |  |  |
| Not married | 704 | 33.3 | 158 | 31.2 | 30 | 22.2 | 0.1 |
| Married | 1176 | 55.6 | 293 | 57.8 | 86 | 63.7 |  |
| Divorced or widower | 235 | 11.1 | 56 | 11 | 19 | 14.1 |  |
| Education |  |  |  |  |  |  |  |
| Elementary or middle school | 311 | 14.7 | 80 | 15.8 | 61 | 45.2 | <0.001 |
| High school | 1122 | 53 | 253 | 49.9 | 45 | 33.3 |  |
| Master degree or higher | 682 | 32.2 | 174 | 34.3 | 29 | 21.5 |  |
| Smoking |  |  |  |  |  |  |  |
| Not smoker | 1186 | 56.1 | 280 | 55.2 | 74 | 54.8 | 0.108 |
| Ex-smoker | 427 | 20.2 | 87 | 17.2 | 34 | 25.2 |  |
| Smoker | 502 | 23.7 | 140 | 27.6 | 27 | 20 |  |
| Structured physical activity |  |  |  |  |  |  |  |
| No | 1342 | 63.5 | 308 | 60.7 | 94 | 69.6 | 0.152 |
| At least 2h/week | 773 | 36.5 | 199 | 39.3 | 41 | 30.4 |  |

Medians were compared using the Kruskal-Wallis test followed by Dunnett's test. Different subscript letters indicate statistically significant differences between groups.

Frequencies and proportions were compared between groups using Chi squared test.

* Body composition and metabolic status information was available for a different number of women.

Table S2: Dietary habits of recruited women

|  | N | % |
| --- | --- | --- |
| Olive oil as main culinary fat |  |  |
| No | 47 | 2.2 |
| Yes | 2068 | 97.8 |
| Olive oil |  |  |
| <4 spoons/day | 1310 | 61.9 |
| ≥4 spoons/day | 805 | 38.1 |
| Vegetables |  |  |
| <2 servings/day | 1432 | 67.7 |
| ≥2 servings/day | 683 | 32.3 |
| Fruit |  |  |
| <3 units/day | 1805 | 85.3 |
| ≥3 units/day | 310 | 14.7 |
| Red meat |  |  |
| ≥1 serving/day | 750 | 35.5 |
| <1 serving/day | 1365 | 64.5 |
| Animal fat |  |  |
| ≥1 serving/day | 50 | 2.4 |
| <1 serving/day | 2065 | 97.6 |
| Sugared beverages |  |  |
| ≥1 glass/day | 334 | 15.8 |
| <1 glass/day | 1781 | 84.2 |
| Wine |  |  |
| <3 glasses/week | 1692 | 80 |
| ≥3 glasses/week | 423 | 20 |
| Legumes |  |  |
| <3 servings/week | 2019 | 95.5 |
| ≥3 servings/week | 96 | 4.5 |
| Fish |  |  |
| <3 servings/week | 1921 | 90.8 |
| ≥3 servings/week | 194 | 9.2 |
| Sweets |  |  |
| ≥3 times/week | 961 | 45.4 |
| <3 times/week | 1154 | 54.6 |
| Nuts |  |  |
| <1 serving/week | 1690 | 79.9 |
| ≥1 serving/week | 425 | 20.1 |
| White meat |  |  |
| No | 707 | 33.4 |
| Yes | 1408 | 66.6 |
| Soffritto |  |  |
| <2 times/week | 1237 | 58.5 |
| ≥2 times/week | 878 | 41.5 |
